# The Willingness to Change Risky Health Behaviors among Chinese Rural Residents: What We Learned from a Population-Based Esophageal Cancer Cohort Study

**DOI:** 10.1371/journal.pone.0161999

**Published:** 2016-08-30

**Authors:** Mengfei Liu, Chanyuan Zhang, Hong Cai, Fangfang Liu, Ying Liu, Jingjing Li, Yaqi Pan, Chuanhai Guo, Zhonghu He, Yang Ke

**Affiliations:** Key Laboratory of Carcinogenesis and Translational Research (Ministry of Education), Laboratory of Genetics, Peking University Cancer Hospital & Institute, Haidian District, Beijing, People’s Republic of China; National Health Research Institutes, TAIWAN

## Abstract

**Background:**

The effectiveness of health interventions can be impaired by low socio-economic status and poor living conditions of the target population. However, the specifics of this problem in rural China are still unclear, and appropriate strategies should be explored.

**Methods:**

In 2013, we conducted a questionnaire-based investigation among 410 participants from a population-based esophageal cancer cohort study in rural Anyang, China. Information regarding their demographic characteristics, levels of exposure to four health-risk behaviors, including smoking, alcohol consumption, risky dietary behaviors and poor hygiene, as well as willingness to change these behaviors, and data on potential predictors of willingness to change behaviors were collected.

**Results:**

In this study, 33.3% (23/69), 25.0% (13/52), 60.7% (68/112) and 62.2% (237/381) of respondents reported that they were willing to change smoking, alcohol consumption, risky dietary behaviors and poor hygiene, respectively. Older people had higher exposure levels and less willingness to change these four health-risk behaviors. The levels of these four health-risk behaviors were negatively associated with willingness to change, while faith in people and behavioral change in surrounding people increased willingness to change risky behaviors.

**Conclusions:**

In behavior-intervention-based health-promotion programs in rural China, the elderly and highly exposed populations should be the most difficult part and community- or household-based intervention would be more efficient.

## Introduction

Esophageal cancer (EC) is among the top causes of cancer-related mortality worldwide, resulting in 406,800 deaths annually [1]. In contrast to ECs in Western countries, 90% of EC cases in China are esophageal squamous cell carcinoma (ESCC) [1]. ESCC mortality in the rural Anyang area of Henan Province, located in north central China, is 10-fold higher than the national average [2,3]. In 2007, a population-based prospective cohort study was initiated in rural Anyang, focusing on the association between ESCC and human papillomavirus (HPV) infection [4]. Over 8000 local permanent residents were enrolled, and to date, one baseline and two follow-up endoscopic screenings have been performed at an interval of two-to-three years [4].

It is well established that behavioral factors play a crucial role in the development of most types of cancer [5]. In high-incidence area of China, a series of behavioral and environmental factors have also been proposed to be potentially associated with the occurrence of ESCC, including cigarette smoking [6,7], alcohol consumption [8,9], risky dietary behaviors [10,11], and HPV infection [12,13]. These findings further argue for the primary prevention of ESCC in China via the management of relevant health-risk behaviors in high-risk populations, which was supposed to be cost-effective [14].

However, low socio-economic status (SES) and poor living conditions may impair the effectiveness of behavioral interventions [15]. In a less developed region of China, such as rural Anyang, the education level of residents is extremely low. We have reported that 43% of residents in rural Anyang do not complete primary school and two-thirds of these illiterate residents are female [4]. Moreover, rural populations generally lack self-concern and health consciousness. The majority of our cohort members (approximately 80%) believed that cancer is not a risk for them, and only 10% have ever undergone a physical examination (data not published). This lack of knowledge and health concern contributes to high exposure of health-risk behaviors in this population. For example, 56.1% of males in rural Anyang are current smokers, a level higher than the national average (52.9%) [16]. The alcohol consumption on special occasions is reported to be 202.3 g/day, the highest among 10 areas across China [17]. Given these characteristics, the pattern of health-risk behavioral change and related factors should be investigated to establish appropriate health-intervention strategies in this population.

In the current study, we administered a cross-sectional questionnaire based on our esophageal cancer cohort study in Anyang. We aimed to examine willingness to change risky behaviors (WCRB) for ESCC, including cigarette smoking, alcohol consumption, risky dietary behaviors, and poor hygiene (correlated with HPV infection) in this population and to evaluate the associated factors.

## Materials and Methods

### Study Field and Subjects

In 2007–2009, a population-based esophageal cancer cohort study (AECCS) was initiated in rural Anyang, China [4]. The study design and the eligibility criteria for AECCS participation have been described elsewhere [4]. Briefly, permanent residents aged 25–65 years in 9 villages in rural Anyang were enrolled based on a cluster-sampling method. Each participant was given a unique identification number (ID) and others had no access to information that could identify individual participants during or after data collection. A routine blood test followed by an endoscopic examination and questionnaire-based interview were performed among all consenting respondents. A relatively high response proportion (8638/10772 eligible subjects) was achieved in the baseline investigation. Hengcun, one of 9 target villages in AECCS with approximately 1000 cohort members, was selected for this study. In 2013, 410 residents of Hengcun who had participated in the baseline AECCS investigation were interviewed on a first-come, first-serve basis.

### Questionnaire Survey

As described elsewhere [18], to minimize bias in the questionnaire investigation, we established a “standard protocol” for the face-to-face survey. Participants were firstly asked about their status and exposure level of four health-risk behaviors including cigarette smoking, alcohol consumption, risky dietary behaviors and poor hygiene habits. Participants who reported to have these behaviors were told by the interviewer that these behaviors were not healthy, and they were then asked about their willingness to change these behaviors. Take smoking for example, if someone reported to smoke, our interviewer would tell him: “You are a smoker, smoking is not healthy, do you want to quit it?”. During the questionnaire investigation, participants were not informed that these targeted behaviors were potentially correlated to ESCC in order to maximally avoid an induced positive answer. Standardized explanations for the items in the questionnaire would be provided if the respondent could not understand or respond correctly. Information collected in the questionnaire included:

1. Demographic characteristics, including age, gender and socio-economic status (SES). The SES score for each participant was calculated as the sum of three scored items (5 categories for each item encoded as 1 to 5 from the lowest level to the highest level): education level, type of employment and monthly household income. Next, the scores were categorized into three groups, where participants with a score of 0–5, 6–10 and 11–15 were defined as low SES, middle SES and high SES, respectively.
2. Willingness to change risky behaviors (WCRB). The WCRB for ESCC, including cigarette smoking, alcohol consumption and risky dietary behaviors, was investigated in this study. The WCRB of poor hygiene, which is not directly related to cancer but has been hypothesized to be correlated with HPV infection, was also investigated to provide a validity check for other questions. Risky dietary behavior was defined as often drinking hot beverages and eating dry, preserved, salted and moldy foods, and poor hygiene included not washing one’s face and brushing one’s teeth before sleep, sharing a towel and brush with family members, an interval between baths >15 days in the winter and not washing the genitals before sex.
3. Potential predictors and factors for WCRB, including the exposure level of these behaviors, family history of ESCC, general health and previously diagnosed diseases, and “peer influence” (behavioral change of surrounding people). The level of smoking and alcohol consumption was defined as the product of daily consumption and the number of years, and the levels of risky dietary behaviors and poor hygiene were calculated by counting the number of positive records in the above-defined behaviors. The range of self-reported previously diagnosed diseases included common diseases of the digestive system, cardio-cerebrovascular disease, respiratory disease and diabetes. Psychological factors, such as faith in people, might also play a role in this issue [19]. Thus, the “Faith in People Scale” (Rosenberg 1958) was integrated in the questionnaire, and the score for “Faith in People Scale” was also evaluated as a potential factor (ranging from 0 to 5, which is inversely associated with faith in people).

### Statistical Analysis

The chi-square test was used to detect differences in the distribution of categorical variables among groups. To compare the WCRB proportions among the four targeted behaviors, a univariate logistic regression model integrated in Generalized Estimating Equations (GEE) was used to adjust for within subject correlation [20]. Four sets of univariate and multivariate unconditional logistic-regression models were fitted to identify factors associated with WCRB, including willingness to change smoking, alcohol consumption, risky dietary behaviors and poor hygiene. Next, observations of smoking, alcohol consumption and risky dietary behaviors were combined and defined as “ESCC-risk behavior”. Given that one participant might contribute to multiple observations, univariate and multivariate GEE logistic-regression models were further fitted to evaluate factors related to willingness to change ESCC-risk behaviors [20]. The backward-selection method with a significance threshold of 0.1 was adopted to identify variables included in the final multivariate models. Candidate variables included age, gender, SES, level of the specific health-risk behavior, self-reported previously diagnosed diseases, scores on the Faith in People Scale, behavioral change of surrounding people and family history of ESCC. Age and gender were treated as design variables and forced into all multivariate models.

All statistical analyses were performed using STATA version 11.2 (STATA Corporation, College Station, TX, USA). All tests were two-sided and had a significance level of 0.05.

### Ethics Statement

Research protocols and materials were approved by the Institutional Review Board of the Beijing Cancer Hospital (Peking University School of Oncology), China. Written informed consent was obtained from all individual participants included in the study.

## Results

A total of 410 participants were interviewed in this study. As shown in Table 1, the median age of enrolled participants was 47 years (interquartile range: 40–57 years), and approximately two-thirds (69.0%) of these individuals were female. For socio-economic factors, 84.4% of the participants were engaged in farming at home; 91.0% had an education level of junior middle school or below; and approximately 30% had a household income of <800 RMB per month. Behavioral factors were as follows: 69 (16.8%) and 52 (12.7%) participants were current smokers and alcohol drinkers; 112 (27.3%) reported risky dietary behaviors; and the vast majority (92.9%) of the participants had poor hygiene. Compared to participants in the current study, the 600 non-included sample is more likely to be younger, male, manual or skilled workers and they exhibited higher levels of all four health-risk behaviors.

**Table 1:** Selected demographic and risk-behavior variables among residents included and *NOT* included in the study, 2013.

Variable	Participants included in the study	Participants *NOT* included in the study ^a^	*p*-value ^b^
	n (%)	n (%)	
	N = 410	N = 600	
**Age at enrollment(years)**			
Median (IQR)	47 (40–57)	43 (34–52)	
25–45	182 (44.4)	369 (61.5)	
46–65	228 (55.6)	231 (38.5)	<0.001
**Gender**			
Female	283 (69.0)	254 (42.3)	
Male	127 (31.0)	346 (57.7)	<0.001
**Type of job**			
Farmers	346 (84.4)	317 (52.8)	
Manual workers	38 (9.3)	157 (26.2)	
Skilled workers	13 (3.2)	53 (8.8)	
Self-employed or managers	13 (3.2)	12 (2.0)	<0.001
Unknown	0 (0)	61 (10.2)	
**Education level**			
Primary school or below	187 (45.6)	221 (36.8)	
Junior middle School	186 (45.4)	298 (49.7)	
Senior middle school	33 (8.1)	52 (8.7)	
Vocational school or bachelor degree	4 (1.0)	6 (1.0)	0.152
Unknown	0 (0)	23 (3.8)	
**Household income (per month)** ^**c**^			
≤400 RMB	59 (14.4)	-	
401–600 RMB	32 (7.8)	-	
601–800 RMB	18 (4.4)	-	
>800 RMB	291 (71.0)	-	
Unknown	10 (2.4)	-	-
**Smoking status**			
No	341 (83.2)	389 (64.8)	
Yes	69 (16.8)	211 (35.2)	<0.001
**Alcohol consumption**			
No	358 (87.3)	474 (79.0)	
Yes	52 (12.7)	126 (21.0)	0.001
**Risky dietary behavior**			
No	298 (72.7)	343 (57.2)	
Yes	112 (27.3)	257 (42.8)	<0.001
**Poor hygiene**			
No	29 (7.1)	20 (3.3)	
Yes	381 (92.9)	580 (96.7)	0.007

^a^ Participants NOT included in the study were defined as participants enrolled in the Esophageal Cancer Cohort Study but not investigated in this survey.

^b^ p-values were derived from the chi-square test.

^c^ Income was not investigated in the Esophageal Cancer Cohort Study.

**Fig 1:**
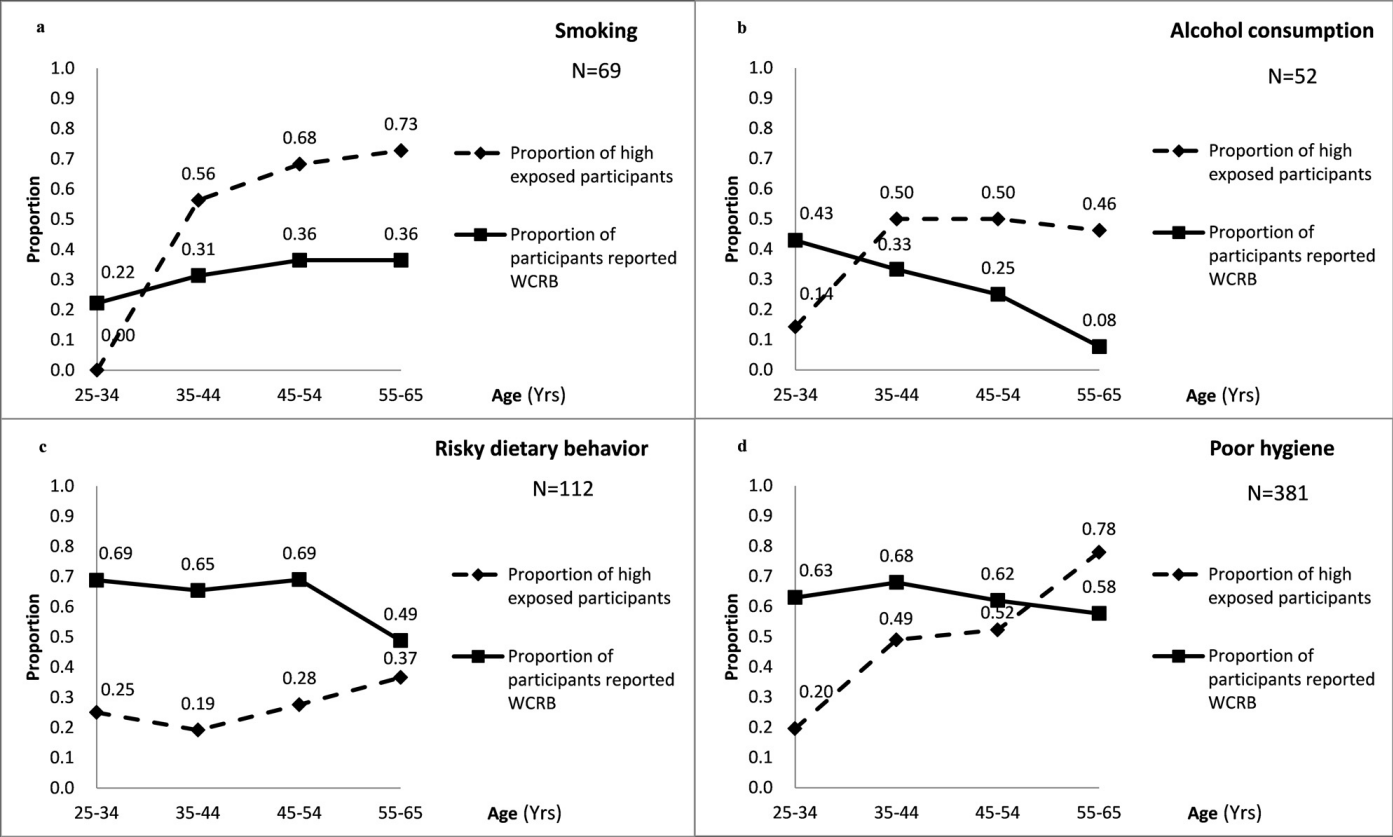
The age distribution of WCRB proportions and exposure level of health-risk behaviors, at 10-year-age intervals*. * Among 410 participants who engaged in the behavior in the current study. ^a^Smoking. ^b^Alcohol consumption. ^c^Risky dietary behavior. ^d^Poor hygiene.

The proportions of participants who reported that they were willing to reduce or quit smoking, alcohol consumption, risky dietary behaviors and poor hygiene were 33.3% (23/69), 25.0% (13/52), 60.7% (68/112) and 62.2% (237/381), respectively. The proportion of people reporting WCRB for smoking and alcohol consumption was significantly lower than the proportion reporting that they were willing to change risky dietary behaviors and poor hygiene (Table 2). A decreasing trend in the proportion of participants declaring WCRB with increasing age was observed for alcohol consumption, risky dietary behaviors and poor hygiene (Fig 1B, 1C and 1D). In contrast, increasing exposure with age was observed for smoking, risky dietary behaviors and poor hygiene (Fig 1A, 1C and 1D), indicating that older people had a higher exposure to most of the health-risk behaviors investigated but tended not willing to change their behaviors.

**Table 2:** WCRB for targeted behaviors among 410 participants who were engaged in the behavior, 2013.

Risk-behavior category	WCRB	*p*-value ^a^
**Smoking (N = 69)**		
No	46 (66.7)	
Yes	23 (33.3)	Ref
**Alcohol consumption (N = 52)**		
No	39 (75.0)	
Yes	13 (25.0)	0.215
**Risky dietary behavior (N = 112)**		
No	44 (39.3)	
Yes	68 (60.7)	<0.001
**Poor hygiene (N = 381)**		
No	144 (37.8)	
Yes	237 (62.2)	<0.001

^a^ *p*-values were derived from a univariate logistic model using the WCRB of participants as the dependent variable and category index (e.g., smoking, alcohol consumption) as the independent variable. GEE was used to adjust for within-subject correlation. Smoking was treated as the reference category.

Risk-factor analysis of ESCC-related risk-behavior showed that participants with high levels of risky behaviors had a significantly decreased WCRB (OR _low vs. high for ESCC-risk behavior_ = 0.38, 95%CI: 0.21–0.68). Less faith in people was also associated with a reduced willingness to change ESCC-risk behaviors (OR _0–2 vs. 3–5 for ESCC-risk behavior_ = 0.62, 95%CI: 0.34–1.11). Behavioral change in surrounding people increased the WCRB (OR _no vs. yes for ESCC-risk behavior_ = 1.71, 95%CI: 0.95–3.71) (Table 3 and S1 Table). Similarly, the WCRB for poor hygiene decreased with increasing exposure and reduced faith in people (OR _low vs. high for inappropriate hygienic behavior_ = 0.54, 95%CI: 0.33–0.87; OR _0–2 vs. 3–5 for inappropriate hygienic behavior_ = 0.51, 95%CI: 0.32–0.81) and increased with behavioral change in surrounding people (OR _no vs. yes for inappropriate hygienic behavior_ = 2.25, 95%CI: 1.32–3.81). High SES was also found to be positively associated with WCRB for hygienic habits (OR _low vs. mid for inappropriate hygienic behavior_ = 1.46, 95%CI: 0.74–2.86; OR _low vs. high for inappropriate hygienic behavior_ = 3.31, 95%CI: 0.97–11.26). The family history of ESCC was associated with neither WCRB for ESCC-risk behavior nor poor hygiene (Table 3).

**Table 3:** Factors associated with WCRB for ESCC-risk behaviors and poor hygiene, 2013*.

Variable	WCRB for ESCC-risk behavior ^a^			WCRB for poor hygiene		
	N = 233 (%) ^b^	Univariate OR ^c^	Multivariate OR ^c^	N = 381 (%)	Univariate OR	Multivariate OR
**Age (continuous)**	-	0.98 (0.96–1.01)	0.99 (0.96–1.01)	-	0.99 (0.97–1.01)	0.99 (0.97–1.02)
**Gender**						
Female	66 (28.3)	Ref	Ref	258 (67.7)	Ref	Ref
Male	167 (71.7)	0.71 (0.40–1.29)	0.81 (0.42–1.55)	123 (32.3)	1.14 (0.73–1.77)	0.89 (0.54–1.46)
**SES**						
Low	24 (10.3)	Ref	NA	43 (11.3)	Ref	Ref
Middle	165 (70.8)	0.77 (0.31–1.90)	NA	303 (79.5)	1.69 (0.89–3.21)	1.46 (0.74–2.86)
High	33 (14.2)	0.85 (0.29–2.51)	NA	25 (6.6)	**4.19 (1.33–13.21)**	3.31 (0.97–11.26)
Unknown	11 (4.7)	-	-	10 (2.6)	-	-
**Exposure of risky behavior**						
Low	138 (59.2)	Ref	Ref	165 (43.3)	Ref	Ref
High	95 (40.8)	**0.35 (0.20–0.61)**	**0.38 (0.21–0.68)**	211 (55.4)	**0.54 (0.35–0.82)**	**0.54 (0.33–0.87)**
Unknown	0 (0)	-	-	5 (1.3)	-	-
**Previously diagnosed diseases**						
No	105 (45.1)	Ref	NA	212 (55.6)	Ref	NA
Yes	128 (54.9)	0.73 (0.42–1.27)	NA	169 (44.4)	0.83 (0.55–1.26)	NA
**Score on the “Faith in People” Scale**						
0–2	148 (63.5)	Ref	Ref	247 (64.8)	Ref	Ref
3–5	85 (36.5)	0.70 (0.40–1.23)	0.62 (0.34–1.11)	134 (35.2)	**0.58 (0.38–0.89)**	**0.51 (0.32–0.81)**
**Behavioral change of surrounding people**						
No	135 (57.9)	Ref	Ref	268 (70.3)	Ref	Ref
Yes	98(42.1)	1.63 (0.93–2.87)	1.71 (0.95–3.07)	113 (29.7)	**2.47 (1.50–4.05)**	**2.25 (1.32–3.81)**
**Family history of ESCC**						
No	182 (78.1)	Ref	NA	296 (77.7)	Ref	NA
Yes	51 (21.9)	0.77 (0.40–1.51)	NA	85 (22.3)	0.78 (0.48–1.28)	NA

* Among 410 participants who were engaged in the behavior. The backward-selection method with a significance threshold of 0.1 was used to identify variables included in the final multivariate models. Confidence intervals that do not overlap the null value of 1 were shown in bold.

^a^ ESCC-risk behavior included smoking, alcohol consumption and risky dietary behavior.

^b^ One participant might contribute to multiple observations in these frequencies.

^c^ The GEE logistic-regression model was used.

## Discussion

We investigated the willingness to change ESCC-associated health-risk behaviors and poor hygiene among rural Anyang residents as part of our ongoing esophageal cancer cohort study. Although self-reported willingness does not itself always accurately reflect real events of behavioral change, a positive response to the question on willingness to change behavior is indicative of a desire to change according to the well-established stages of change model [21]. The results of this study are important to future behavior-intervention-based cancer-prevention programs, as well as other health-education and -promotion efforts in rural China.

According to our investigation, in which health advice was delivered one-on-one along with endoscopic screening or questionnaire administration, approximately one-third and one-quarter of current smokers and alcohol consumers reported that they were willing to reduce or quit smoking and alcohol consumption, while nearly three-fifths reported a positive attitude toward changing risky dietary behaviors and poor hygiene. Generally, the proportions of WCRB for cancer-related behaviors in our population were lower than those in Western populations, among whom studies have reported that 70% of smokers were willing to quit [22], that 81% of alcohol drinkers had a positive attitude toward change [23] and that 86% of participants at high risk of colorectal cancer adhered to diet-change advice [24]. These discrepancies may initially be attributed to the low SES in our rural Chinese population [25]. SES was negatively associated with the establishment of health beliefs and attitudes [15] toward health-risk behaviors. The limited education level of our participants may have resulted in the absence of necessary health knowledge and beliefs and ignorance regarding health conditions, which would further have greatly diluted the health effects of our advice. Moreover, the relatively low income of the rural population would reduce their opportunities to obtain health-related knowledge and information, which could also contribute to the lower proportion responding to our health advice. Second, the method of dispensing health advice could also affect the WCRB of the cohort members. Detailed explanations of the consequences of health-risk behaviors, particularly of the potentially cancer-related behaviors could further improve the willingness to change behaviors among these rural residents. Expectedly, significantly lower proportions of willingness to reduce or quit smoking and alcohol consumption were detected compared to risky dietary behaviors and poor hygiene. This outcome could be explained by the fact that cigarettes and alcohol are addictive substances, which might result in psychological dependence and cause considerable difficulty in quitting [26].

For most of the targeted ESCC-risk behaviors and poor hygiene investigated in the current study, exposure level was positively correlated with age, while WCRB was negatively correlated with age. This finding indicated that elderly people tended to have more health-risk behaviors but less willingness to change them. And this work further revealed discrepancies between the old and young generations in this rural population. With the socio-economic development of China in recent decades, substantial changes have taken place in terms of education levels and concepts of health and lifestyle. For example, we identified significant differences in demographic variables (e.g., education level, type of job) and behavioral features (e.g., conservation on sex, hygienic habits) between old and young males in the same population [27]. Older residents, with higher levels of cumulative exposure, tend to have higher levels of health-risk behaviors but are usually less willing to change these behaviors. Explanatory factors include their limited ability to understand and accept health advice and their traditional and outdated health-related beliefs.

We found that the exposure level of health-risk behavior was strongly negatively associated with the WCRB for all targeted behaviors. This finding might be the result of behavioral inertia resulting from their long-term unconscious habits and limited health knowledge and beliefs, as well as addiction. Besides, our results indicated that behavioral change of surrounding people was positively associated with WCRB in this population. Unlike Western people, people in rural China have less sense of privacy and more concern regarding what people think of them. They tend to share their experiences in the cohort study with others during the investigation and at home. People with unhealthy behaviors or habits would have a greater chance of being influenced by their living environment, such as expostulation from their friends, neighbors and family members. A circumstantial evidence of this assumption is that participants who have more trust in other people are more likely to report positive attitude to change health-risk behaviors in this study. Similar results were detected when willingness to change ESCC related behaviors and poor hygiene was evaluated separately, which indicates that the willingness to change health-risk behaviors in this rural population might have common determinants, independent of whether the behaviors are cancer-related.

Several limitations of this study should be noted. First, the participants enrolled in this study were not a random sample of the population due to the first-come first-serve study design, which was mainly because that young male adults in rural China were more likely to work outside their hometown and were less likely to be interviewed in this study [28]. Although the non-included young individuals reported higher proportions of all four health-risk behaviors than included ones, they tended to have low exposure levels and high WCRB proportions of health-risk behaviors according to our results, which indicated that there should be an under-estimation of the percent of people expressing willingness to change a risky health behavior. Second, participants would probably have the desire to please the interviewer during the face-to-face investigation, which conversely could result in an over-estimation of WCRB. Synthesizing these two aspects of limitations, the direction of bias in the current study could not be accurately predicted. Prospective study on the basis of up to 10-year follow-up data of AECCS has been initiated which would dynamically observe the actual change of risk behaviors among these cohort members and systematically address this issue. Despite these, this pilot study demonstrated a series of characteristics of health-risk behavior as well as WCRB in this population, and provided crucial basis for developing appropriate strategy of behavior-intervention-based health education and promotion programs in rural China.

## Conclusions

When considering an appropriate health-intervention strategy in a less developed area, we would face two major difficulties: first, the large demand and limited resources; second, the need to identify an applicable and efficient method for a health intervention among uneducated and conservative villagers. The results of our study emphasize the importance of designing a localized education model to ensure an ideal intervention effect. The results revealed that elderly people and people with high exposure level would be the most difficult part of health promotion programs in rural China due to their lower willingness to change health-risk behaviors. On the other hand, young people with a lower exposure level should be the focus of public health efforts since one at a more advance stage of readiness to change may be more likely to change their behavior given limited resources. In addition, the household or community other than the exposed individuals should be considered as the basic unit of intervention, and a mass media approach would be most effective, creating a climate of disapproval for health-risk behaviors such as smoking and drinking. Taken together, these findings may be of importance when implementing behavioral intervention programs in resource-limited settings such as rural China.

## Supporting Information

S1 Table Factors associated with WCRB for smoking, alcohol consumption and risky dietary behavior, 2013. (DOCX)
