# Supplementary material for: The Willingness to Change Risky Health Behaviors among Chinese Rural Residents: What We Learned from a Population-Based Esophageal Cancer Cohort Study
Source: PLoS One. 2016 Aug 30;11(8):e0161999. doi: 10.1371/journal.pone.0161999 (PMC5004976; doi:10.1371/journal.pone.0161999)
Supplement: S1 Table — (DOCX) [file pone.0161999.s001.docx]

| **S1 Table.** Factors associated with WCRB for smoking, alcohol consumption and risky dietary behavior, 2013 | | | | | | | | | | | |  |
| --- | --- | --- | --- | --- | --- | --- | --- | --- | --- | --- | --- | --- |
| **Variable*^*^*** | WCRB for smoking | | |  | WCRB for alcohol consumption | | |  | WCRB for risky dietary behavior | | | |
|  | N=69 (%) | Univariate OR | Multivariate OR |  | N=52 (%) | Univariate OR | Multivariate OR |  | N=112 (%) | Univariate OR | Multivariate OR | |
| **Age (continuous)** | - | 1.01 (0.96-1.06) | 1.01 (0.95-1.07) |  | - | 0.95 (0.89-1.01) | 0.95 (0.88-1.01) |  | - | 0.97 (0.94-1.00) | 0.97 (0.93-1.00) | |
| **Gender** |  |  |  |  |  |  |  |  |  |  |  | |
| Female | 0 (0) | Ref | Ref |  | 3 (5.8) | Ref | Ref |  | 63 (56.3) | Ref | Ref | |
| Male | 69 (100.0) | - | - |  | 49 (94.2) | 0.65 (0.05-7.80) | 0.95 (0.07-13.71) |  | 49 (43.7) | **2.68 (1.20-5.99)** | **2.94 (1.27-6.79)** | |
| **SES** |  |  |  |  |  |  |  |  |  |  |  | |
| Low | 7 (10.1) | Ref | Ref |  | 2 (3.8) | Ref | NA |  | 15 (13.4) | Ref | NA | |
| Middle | 47 (68.1) | 0.32 (0.06-1.61) | 0.23 (0.04-1.40) |  | 37 (71.2) | NE | NA |  | 81 (72.3) | 1.27 (0.42-3.85) | NA | |
| High | 12 (17.4) | 0.25 (0.03-1.82) | 0.13 (0.01-1.21) |  | 12 (23.1) | NE | NA |  | 9 (8.0) | NE | NA | |
| Unknown | 3 (4.3) | - | - |  | 1 (1.9) | - | - |  | 7 (6.3) | - | - | |
| **Exposure of risky behavior** |  |  |  |  |  |  |  |  |  |  |  | |
| Low | 29 (42.0) | Ref | Ref |  | 29 (55.8) | Ref | Ref |  | 80 (71.4) | Ref | Ref | |
| High | 40 (58.0) | 0.41 (0.15-1.14) | **0.25 (0.07-0.97)** |  | 23 (44.2) | 0.29 (0.07-1.20) | 0.22 (0.05-1.06) |  | 32 (28.6) | 0.45 (0.20-1.04) | 0.48 (0.20-1.14) | |
| Unknown | 0 (0) | - | - |  | 0 (0) | - | - |  | 0 (0) | - | - | |
| **Previously diagnosed diseases** |  |  |  |  |  |  |  |  |  |  |  | |
| No | 31 (44.9) | Ref | NA |  | 24 (46.2) | Ref | NA |  | 50 (44.6) | Ref | NA | |
| Yes | 38 (55.1) | 0.49 (0.18-1.36) | NA |  | 28 (53.8) | 0.43 (0.12-1.57) | NA |  | 62 (55.4) | 0.91 (0.42-1.95) | NA | |
| **Score on the “Faith in People” Scale** | |  |  |  |  |  |  |  |  |  |  | |
| 0-2 | 44 (63.8) | Ref | NA |  | 31 (59.6) | Ref | Ref |  | 73 (65.2) | Ref | NA | |
| 3-5 | 25 (36.2) | 0.68 (0.23-1.98) | NA |  | 21 (40.4) | 0.35 (0.08-1.47) | 0.22 (0.04-1.13) |  | 39 (34.8) | 0.89 (0.40-1.98) | NA | |
| **Behavioral change of surrounding people** |  |  |  |  |  |  |  |  |  |  |  | |
| No | 44 (63.8) | Ref | NA |  | 29 (55.8) | Ref | NA |  | 62 (55.9) | Ref | NA | |
| Yes | 25 (36.2) | 2.77 (0.98-7.83) | NA |  | 23 (44.2) | 1.11 (0.31-3.91) | NA |  | 49 (44.1) | 1.50 (0.69-3.24) | NA | |
| **Family history of ESCC** |  |  |  |  |  |  |  |  |  |  |  | |
| No | 52 (75.4) | Ref | NA |  | 39 (75.0) | Ref | NA |  | 91 (81.3) | Ref | NA | |
| Yes | 17 (24.6) | 0.34 (0.09-1.34) | NA |  | 13 (25.0) | 0.87 (0.20-3.81) | NA |  | 21 (18.8) | 1.37 (0.50-3.72) | NA | |
| ^*^ Among 410 participants who were engaged in the behavior. The backward-selection method with a significance threshold of 0.1 was used to identify variables included in the final multivariate models. Confidence intervals that do not overlap the null value of 1 were shown in bold. | | | | | | | | | | | |  |
